# Study of Multi-Armed Bandits for Energy Conservation in Cognitive Radio Sensor Networks

**DOI:** 10.3390/s150409360

**Published:** 2015-04-21

**Authors:** Juan Zhang, Hong Jiang, Zhenhua Huang, Chunmei Chen, Hesong Jiang

**Affiliations:** 1 Open Fund of Robot Technology Used for Special Environment Key Laboratory of Sichuan, Mianyang 621010, China; E-Mails: zhangjuan@swust.edu.cn (J.Z.); jianghong@swust.edu.cn (H.J.); chenchunmei@swust.edu.cn (C.C.); jianghesong@wust.edu.cn (H.J.); 2 School of Information Engineering, Southwest University of Science and Technology, Mianyang 621010, China; 3 School of Electronics and Information, Tongji University, Shanghai 201804, China

**Keywords:** packet size adaptation, energy efficiency, multi-armed bandits, cognitive radio sensor networks

## Abstract

Technological advances have led to the emergence of wireless sensor nodes in wireless networks. Sensor nodes are usually battery powered and hence have strict energy constraints. As a result, energy conservation is very important in the wireless sensor network protocol design and the limited power resources are the biggest challenge in wireless network channels. Link adaptation techniques improve the link quality by adjusting medium access control (MAC) parameters such as frame size, data rate, and sleep time, thereby improving energy efficiency. In this paper we present an adaptive packet size strategy for energy efficient wireless sensor networks. The main goal is to reduce power consumption and extend the whole network life. In order to achieve this goal, the paper introduces the concept of a bounded MAB to find the optimal packet size to transfer by formulating different packet sizes for different arms under the channel condition. At the same time, in achieve fast convergence, we consider the bandwidth evaluation according to ACK. The experiment shows that the packet size is adaptive when the channel quality changes and our algorithm can obtain the optimal packet size. We observe that the MAB packet size adaptation scheme achieves the best energy efficiency across the whole simulation duration in comparison with the fixed frame size scheme, the random packet size and the extended Kalman filter (EKF).

## 1. Introduction

Sensing, processing and communication are integrated into a tiny wireless sensor network (WSN) device. They are used in inaccessible environments and maintenance is typically inconvenient or impossible because wireless sensor networks are robust and distributed. WSNs are widely used in residential, industrial, and environmental monitoring. WSNs are being more and more used in outdoor environments to monitor weather conditions, the natural habitat, the scene of disasters, ecological systems, nuclear accident sites, *etc.* Compared to traditional wired networks, WSNs are relatively simple and inexpensive. In addition, such networks can be easily extended by simply adding more devices without reworking and reconfiguring the whole network. The most direct application of sensor networks is in remote environmental monitoring. For example, large numbers of sensors can be deployed in a remote forest area to immediately report any incident such as a fire. Ideally, the sensor node of a set of batteries can run for a year. Considering the cost of sensor nodes (SNs), discarding sensor nodes without electricity is not feasible, and it may be impossible to replace the batteries of SNs. Therefore, there is a huge demand for an energy conservation scheme to reduce energy consumption and prolong the lifetime of wireless sensor nodes [1,2].

At present, wireless microsensor nodes are about the size of a quarter dollar. Meeting the energy demands is the biggest challenge in WSNs. Therefore, the sensor network platform must provide an energy-efficient protocol to greatly reduce power consumption. The energy efficiency is defined as the ratio between the number of data transmissions and the energy consumption. Table 1 shows the power measurement of a typical sensor node in an active, sleep, transmit, and receive modes [3].

**Table 1:** Power measurements of a sensor node [3].

State	Energy Consumptions
Transmit	10.8 mA (radio)
	8 mA (cpu)
Idle/Receive	7.5 mA (radio)
	8 mA (cpu)
Sleep	1 uA (Radio)
	1 uA (cpu)

The wireless network is time-varying. If a number of error packets are dropped at the receiving end, this will result in retransmissions resulting in a waste of bandwidth and finally lead to energy consumption. Therefore, reducing the number of retransmissions can help minimize the energy consumption. The main reason for his is framing errors. There are three main types: interference, slow fading and fast fading. Interference is due to white Gaussian noise or other users using the same frequency channel. The path loss and shadows are the main reason for slow fading. Fast fading is mainly due to spread delays or Doppler frequency. As mentioned above, the retransmission is the main cause of wasted energy [4,5,6].

This paper presents a link adaptive mechanism to improve the energy efficiency and extend the whole network life. Our focus is to improve the MAC layer protocol. MAC is in charge of transmitting data over the physical channel. Because the frame error rate is dependent on the packet size, we need to revise the amount of data sent according to the channel quality at any time. We use variable frame sizes to replace the fixed frame size. Variable frame size can improve throughput; if the network state is good, sensor nodes can transmit big data packets. Otherwise, they transmit small data packets if the network state is bad. According to the channel quality we use a multi-armed bandit (MAB) model to optimize the frame size to improve the energy efficiency. We introduce bandwidth estimation based on ACK interval to evaluate the wireless channel quality and use the multi-armed bandit (MAB) model to find the optimal packet size for data transmissions. This paper proposes three important aspects:

- The frame size adaptive adjustment replaces the fixed frame transmission according to the channel quality.
- MAB is applied to predict the optimal frame size according to the channel quality.
- Bandwidth estimation based on the ACK interval is used for tracking the wireless channel quality.
- The MAB scheme is compared with the extended Kalman filter (EKF) [3], which is better in the current research of adaptive packet length.

The rest of the paper is organized as follows: in Section 2, we discuss related work. Then, in Section 3, we formally describe the system model. Section 4 outlines MAB and the packet size adaption and then analyses its performance in Section 5. A simulation is described in Section 6. We comment on future work in Section 7.

## 2. Related Work

### 2.1. Energy Efficiency

There are many studies that have sought to solve the energy efficiency problem in wireless sensor networks. Gao *et al.* [7] formulated an optimal model to minimize the energy per bit for energy-efficient spectrum access of each single user. Chen and Zhao [8] considered the channel state and residual energy for MAC protocols.Wang *et al*. [9] and Yang *et al*. [10] proposed a realistic energy consumption model for sensor nodes to reduce the conditions in data transmissions. However, these studies were based on ordinary WSNs and they did not need to consider the time-varying and bursty wireless channel quality. A few recent papers integrate cognitive radio into the wireless sensor network. Akan *et al.* [11] and Goh *et al.*[12] introduced the main advantages and challenges of the CRSN. Vijayand *et al.* [13] provided a framework of cognition in sensor networks. Zhang *et al.* [14] and Maleki *et al.* [15] proposed reliable and energy efficient techniques for CRSNs. Liang *et al.* [16] analyzed the CRSN delay and supports time-varying channel. Tian *et al.* [17] and Zhu *et al.* [18] developed a novel algorithm with power allocation, which maximizes channel utilization and minimizes power consumption. He *et al.*[19] minimized the cost function, and consider both the energy consumption and the packet loss rate. Naqvi *et al.* [20] and Sankarasubramaniam *et al.* [21] explored packet optimization and a fixed frame size. Mastronarde *et al.* [22] and Woo *et al.* [23] proposed adaptive data transmission rate for WSNs. Ci *et al.* [24,25] proposed the Kalman filter for predicting the optimal frame size wireless networks. The objective of this research is to adapt time-varying wireless networks, but it is difficult to get the instantaneous quality of the channel.

### 2.2. Multi-Armed Bandits

Multi-armed bandits model is composed of an M arms machine. Each arm can get rewards when drawing the arm, and the arm pulling distribution is unknown. The arm is drawn and gets a reward at each time step. Choosing which of these arms to draw and maximize the sum of the rewards is the target. Ho *et al.* [26] and Bubeck *et al.* [27,28] proposed that it didn’t initially know the reward value before pulling a arm and it must to learn by observation. The goal is to maximize returns and select different actions through the amount of order. Auer *et al.* [29] proposed that the goal is to find the arm as early as possible and keep using it to reach the highest reward. However, the MAB model shows an incomplete description, which is an agent of sequential decision-making problems in many real worlds. To this end, all kinds of related models have recently been studied by Beygelzimer *et al.* [30], In particular, within budget-limited MABs, each action has a different reward and is constrained by the total energy. To solve this problem, Tran-Thanh *et al.* [31] proposed some efficient algorithms which are the unbounded ε-first and KUBE. As we will explain later, the bounded ε-first method depends on the highest rewards in the exploration phase, However, we will discuss in theory and in practice the uniform exploration phase.

## 3. System Model

**Figure 1:**
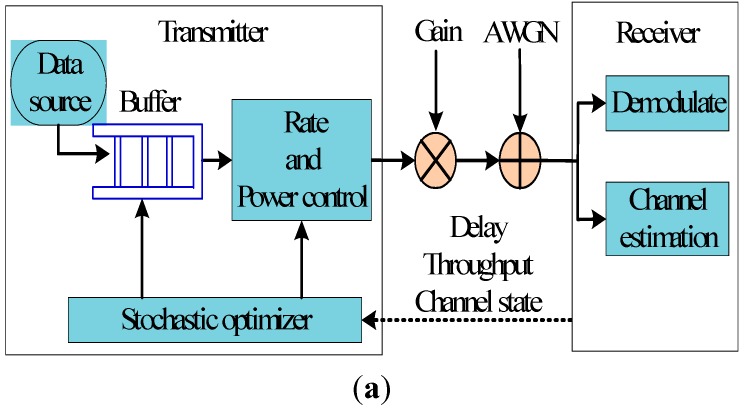

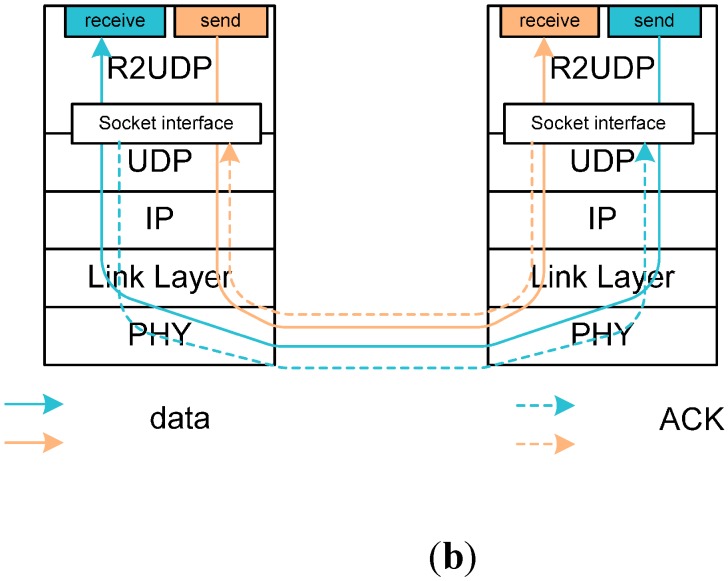
(**a**) Wireless transmission system; (**b**) Logical Reliable Channel.

Figure 1 illustrates a wireless transmission system. We consider a point to point system with a single user which includes a transmitter and receiver. It transmits data from the finite buffer queue over a time-varying channel. At the same time, the transmission time is divided into equal time slots Δ*t*, and the discrete time interval is represented as [*n*Δ*t*, (*n*+1)Δ*t*] (n is the time slot). We assume that the system state is time-varying in each time slot. According to the delay feedback, the receiver can obtain the throughput and channel state information, and the transmitter can adaptively adjust the transmission rate and transmission power [32].

### 3.1. Network Configuration

**Figure 2:**
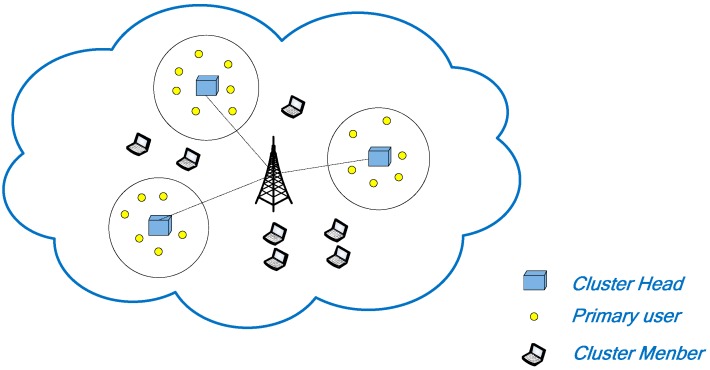
A cognitive radio sensor networks with the primary user.

Figure 2 shows the scenario where the primary user (PU) and the cognitive radio sensor network (CRSN) coexist in the same area. We assume that the transmission power of the primary user is very high when it is in the static or mobile condition. Sensors are very little mobile in the range of a cluster. The sensor and PU transmission should not cause any interference. This can be achieved by power control of the sensors, which is beyond the scope of this paper. Also, the sensor should immediately stop the transmission data when PU signals are detected. An *ad hoc* CRSN has two disadvantages. First of all, it is energy constrained, thereby limiting the transmission range of the sensor. Second, it plays an essential role in the network-wide common control channel of a general CRSN. However low power consumption and a large environment area are not feasible. As such, we propose a multi-channel cognitive radio sensor network structure. The structure includes a cluster head (CH) and a set of cluster members (CMs). The CH is an high energy source with a high performance sensor. CMs are regular low-power sensors. The CH performs spectrum sensing and allocation. To make the spectrum allocation decisions, the parameters of the PU are estimated based on the sample sequence. Then, the CH makes a decision on the packet size and channel assignment based on the estimation results [33,34,35].

### 3.2. Physical Layer Model

**Figure 3:**
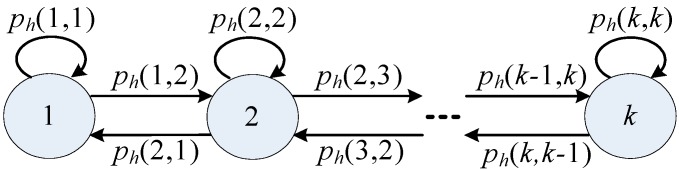
FSMC model.

We consider the Rayleigh fading channel model which is a discrete time block with additive white Gauss noise. Its power spectrum density is *N*_0_/2 . The wireless channel bandwidth is *W*. In this paper, we use a Finite State Markov Channel (FSMC) in order to describe the wireless channel. As shown in Figure 3, there are *k* channel states and each can be transfered to its adjacent states with corresponding probabilities.

The signal-to-noise ratio (SNR) φ is received instantaneously and exponentially distributed in the Rayleigh fading channel. The probability density function is:

(1) $$f_{\varphi }(\varphi )=\varphi_{0}\exp(-\frac{\varphi }{\varphi_{0}})$$

where φ_0_ = E[φ] is the average gain of the channel. If the received SNR is in the interval [φ_k_, φ_k + 1_], the channel is in state h_k_. N(φ) is the level crossing rate (LCR). It is given by:

(2) $$N(\varphi )=\sqrt{\frac{2\pi \varphi }{\varphi_{0}}}\cdot f_{d}\cdot \exp(-\frac{\varphi }{\varphi_{0}})$$

where, *f_d_* is the maximum Doppler frequency. Hence, the state transition probability can be obtained by the following formula:

(3) $$\left\{ \begin{array}{l} p_{h}(k,k+1)=N(\varphi_{k+1})\cdot \Delta t/\pi_{k}\;，1\;\leq k\leq K-1\\ p_{h}(k,k-1)=N(\varphi_{k})\cdot \Delta t/\pi_{k}\;，2\leq k\leq K\\ p_{h}(k,k)=1-p_{h}(k,k+1)\;,k=1\\ p_{h}(k,k)=1-p_{h}(k,k-1)\;,k=K\\ p_{h}(k,k)=1-p_{h}(k,k+1)-p_{h}(k,k-1)\;,k\neq 1,K \end{array}\right.$$

where, the steady state probability *π_k_* is given by:

(4) $$\pi_{k}=\int_{\varphi_{k}}^{\varphi_{k+1}}f_{\varphi }(\varphi )d\varphi =\exp(-\frac{\varphi_{k}}{\varphi_{0}})-\exp(-\frac{\varphi_{k+1}}{\varphi_{0}})$$

### 3.3. MAC Layer Model

**Figure 4:**
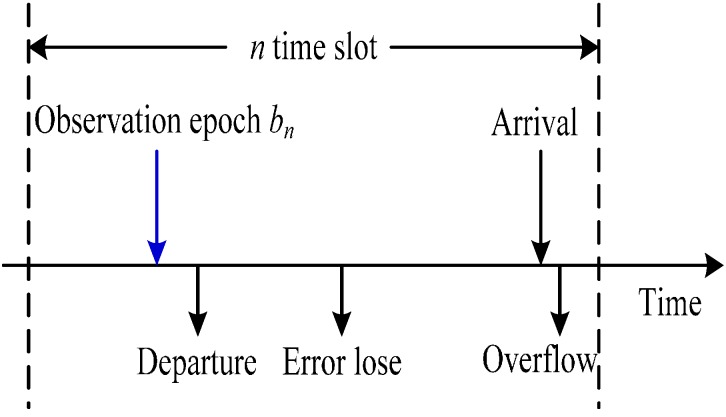
Buffer timing diagram.

In Figure 4, the transmission buffer is a first in first out (FIFO) queue. The transmitter receives and stores ln packets in the finite buffer ,then sends some packets from the buffer in the n-th time slot,. We assume that the traffic arrival distribution follows an independent and identical distribution (i.i.d) during each slot and the packets arrival follows a Poisson process with rate λ. Hence, when one packet arrives, the probability density is denoted as:

(5) $$p_{l}(l)=\frac{\lambda^{l}\cdot \exp(-\lambda )}{l!}$$

Afterwards, we define that the backlog is denoted by b∈[0, B] at the transmitter buffer. B is the capacity of the finite buffer. Each packet contains L bits.When the buffer is full, the arriving packets will be dropped. The packet zn will be sent in slot n at the transmitter, where zn∈{0, 1, …, B}. The packets are affeced by the Bit Error Ratio (BER) and smaller than zn, *i.e.*, fn(BERn, zn)≤zn when received at the receiver. fn is represented by a binomial distribution when packet losses are independent:

(6) $$p^{f}(f_{n}|BER_{n},z_{n})=bin(z_{n},1-PER_{n})$$

where *PER* is the packet error ratio, which meets $PER_{n}=1-{\left(1-BER_{n}\right)}^{l_{n}}$ *b_int_* is the initial buffer state. *b_n_* is the buffer state at the *n*-th slot. Therefore, the buffer state can evolve recursively at the transmitter as follows:

(7) $$\left\{ \begin{array}{l} b_{0}=b_{\mathrm{int}}\\ b_{n+1}=\min(b_{n}-z_{n}+l_{n},B) \end{array}\right.$$

### 3.4. Dynamic Power Management Model

We assume that the wireless card can switch to a low-power state to reduce power consumption. The card has two power management states, *i.e.*, *X* ∈ {*on*, *idle*}. Furthermore, the power state can be switched between *on* and *idle* by the corresponding actions which is in the set *Y=*{*s_on*, *s_idle*}. The corresponding power overheads are defined as *P_on_* and *P_idle_* in the *on* and *idle* states, respectively. When the state transitions from *on* to *idle* or *vice versa*, *P_tr_* is the power consumption. In the *n*-th slot, the packet throughput is *z*, and the required power is as follows:

(8) $$\rho ([h_{n},x_{n}],BER_{n},y_{n},z_{n})=\left\{ \begin{array}{l} P_{idle\;}\;,if\;x_{n}=idle,y_{n}=s\_idle\\ P_{on}+P_{tx}(h_{n},BER_{n},z_{n})\;,if\;x_{n}=on,y_{n}=s\_on\\ P_{tr}\text{ ​​ },otherwise \end{array}\right.$$

where *x_n_* is the power management state, *y_n_* is the power management action, *P_tx_* is the transmission power, *h_n_* is the channel state. *u is* the number of symbols per slot. The transmission power is given by:

(9) $$P_{tx}\geq \frac{W\cdot N_{0}}{h_{n}}\cdot \frac{-\log(5\cdot BER_{n})\cdot (2^{\frac{z_{n}\cdot L}{u}}-1)}{1.5}$$

**Figure 5:**
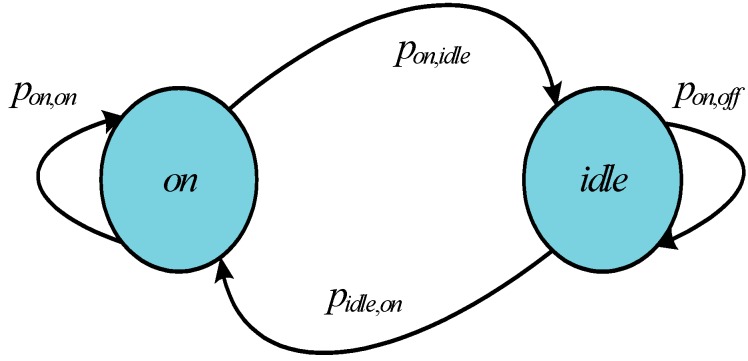
State diagram of the power model.

We assume that the delay of the power state switching from one state to another is negligibly small when the power management operation is implemented. Let *P_x_*(*y*) = [*p*(*x’*|*x*, *y*)]_*x*,*x’*_ represent the transition probability matrix. It means that the power state is switched from *x* to *x’* when the power management action is *y*. In Figure 5, the power management states can be modeled as a constrained Markov chain with transition probabilities.

### 3.5. Energy Consumption Model

**Figure 6:**
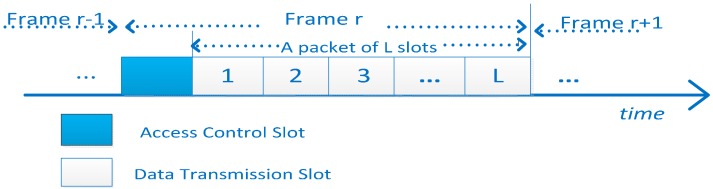
the frame time-slotted structure.

As shown in Figure 6, the system has L + 1 time slots in each frame. It contains an access control slot and L data transmission slots. The duration is T units. CH and CM control information is exchanged through access control slots. A packet need a frame transmission, so the packet size is B × T × L. The cluster operation determines B and T. In order to efficiently utilize the battery of each sensor, L is adaptable according to the time-varying channel conditions. The CMs transmit data packets according to control information in data transmission slot [36,37,38].

Since the CH has a long-duration power supply, it is not subject to energy constraints. This paper considers only the energy consumption of CMs when they exchange control message and transfer data.

Each sensor has the same initial energy E_in_ which is obtained from a non-rechargeable battery in a cluster. Our work is only concerned about the impact of dynamic spectrum access protocol for energy efficiency, so that the communication channel only considers the impact of the path loss. The energy consumption is $E_{cir}\text{+}\varepsilon d_{i}^{\alpha }$ in data transmission. ɛ is power amplifier at the receiving end. The distance between the cluster head and the cluster members is *d*. E_cir_ is the energy consumption of the circuit. E_cir_ and ɛ are computed by per bit. α is the path loss coefficient. Its value depends on the channel quality. L represents the continuous transmission data of the number of slots [39]. Hence, the total energy consumption is:

(10) $$E_{i}^{tr}(l)=(E_{cir}+\varepsilon d_{i}^{2})\times B\times T\times l$$

where E_cir_ is nJ/bit. ɛ is in pJ/bit/m^2^, B is the transmission rate (bit/s), T is the slot length (seconds). Since CMs receive the CH messages, reception energy consumption also needs to be considered. The expression is as follows:

(11) $$E_{i}^{TV}(l)=E_{cir}\times B\times T\times l$$

##  4. The Model of Packet Size Adaptation Using Bounded Multi-Armed Bandits

This section introduces the bounded multi-armed bandits model, then describes the packet size adaptation problem. Finally, we show how to map packet size adaptation to MAB by exploration and exploitation.

### 4.1. Bounded Multi-Armed Bandits

The MAB proposed is composed of K arms, represented as 1,2,⋯,N. An arm is chosen from a non-empty subset S(t)⊆{1,2,⋯,N} to draw at each time step, and we pay a drawing cost ci. The cost budget is B. It means that the total cost is no more than this budget constraint B. When a nonnegative return is received, its distribution is associated with a particular arm. We assume that each arm support has a limited return distribution, because the reward value is usually restricted in an actual application. When the arm i is drawn, the agent receives the mean value of the reward μi. Maximizing the total of the rewards that one gets from drawing the arm is the goal, but the agent does not have the original knowledge μi of each arm, so it is necessary to learn these values to select a strategy to maximize total return. In view of this, the goal is to find the arm whose rewards is the most to draw, whose total reward can achieve the maximum expectation, no more than B [31].

Formally, A is the arm-pulling algorithm, which can get the finite sequence. NBi(A) is the random variable which is the number of the arm i pulled by A. B is the budget limit. NBi(A) is the random variable, because A depends on the reward is observed. Therefore, we have:

(12) $$N_{\text{i}}^{B}(A)=\sum_{t}I\{i\in S^{A}(t)\}$$

where $S^{A}(t)$ is the subset where *the agent* chooses the arm i to draw. $I\{i\in S^{A}(t)\}$ is the indicator function. In order to ensure that the sequence of total cost is no more than B, we have:

(13) $$P(\sum_{i=1}^{N}N_{\text{i}}^{B}(A)c_{i}\leq B)=1$$

where P (•) represents the probability. Furthermore, it assumes that the agents draw each arm number which is no more than L_i_. That is:

(14) $$\forall \text{i: P(}N_{\text{i}}^{B}(A)\leq L_{i}\text{)=1}$$

Now, let ${\text{G}}^{B}\text{(A)}$ be the total return which is got by using the A algorithm to draw the arms and the cost is no more than B. The expectation value of G^B^ (A) is:

(15) $${\text{E[G}}^{B}\text{(A)]=}\sum_{i=1}^{N}E[N_{\text{i}}^{B}(A)]\mu_{i}$$

Then, $A^{*}$ represents the optimal algorithm. It maximizes the total return:

(16) $$A^{*}=\arg\;\underset{A}{\max}\sum_{i=1}^{N}E[N_{\text{i}}^{B}(A)]\mu_{i}$$

In order to achieve the optimal algorithm A*, we have to understand in advance the value of μi, which is not saved in our case. Therefore, A* is on behalf of the theory of the optimization algorithm which could not be achieved. However, the regret is defined as the difference between the expected total return and the optimal value A* [40]. That is:

(17) $$R^{B}(A)=E[{\text{G}}^{B}{\text{(A}}^{*}\text{)}]-E[{\text{G}}^{B}\text{(A)}]$$

Here the purpose is to get a sequence of arm pulls which minimizes the regret of the above definition. It is a bounded multi-armed bandits problem. Since we limit $L_{i}=\infty \;|S(t)|=1$, we get the budget restricted MAB. Moreover, when setting B=∞, we can obtain the standard MAB model.

### 4.2. Packet Size Adaptation

The nature of the wireless channel is time-varying. It is very inefficient when a fixed frame size is used. As previously discussed, a variable frame size minimizes the error caused by the frame rate of a large data packet transmission through a bad channel quality. If the channel quality is better, a large packet size is sent. The variable frame size reduces the number of retransmissions, increases the goodput of the system and saves energy. The paper finds the optimal size by MAB according to the different channel quality. The frame size is a local optimum, because the number of users and channel quality change, making the network environment change too [41,42,43,44].

In order to make use of multi-armed bandits to find the optimal frame size according to the channel quality, we need to develop exploration and exploitation. Our goal is to maximize the goodput of the networks. The channel goodput considered for developing exploration and exploitation which depends on the packet size, collision rate, and data rate, the delay of the protocol and the quality of the channel. The following equation gives the relationship between throughput and the frame size [3]:

(18) $$\rho =\frac{LR}{\left((L+H_{MAC})({\left(1-P_{error}\right)}^{-(L+H_{MAC})}+N)+(T+D+H_{PHY}+O_{protocol})(({\left(1-P_{error}\right)}^{-(L+H_{MAC})}+N)R+ACK{\left(1-P_{error}\right)}^{-L_{ACK}}+O_{ACK}({\left(1-P_{error}\right)}^{L_{ACK}}*R+N*L_{collision})\right)}$$

L: a frame size
L_ACK_: the acknowledgment frame length
L_collision_: the average collision length
R: the transmission of data rate
H_MAC_: the MAC protocol of a frame header
H_PHY_: the PHY layer of a frame header
N: average collisions number between two renewal sensor
T: average backoff time slots under a certain channel
D: Distributed Inter-frame Spacing(DIFS)
P_error_: bit error probability in the case of known channel quality
O_ACK_: the overhead of the acknowledgment
O_ptotocol_: the MAC and PHY protocol process delay overhead

Firstly, from the PU behavior indicated in Equation (19), we find that the probability that the packet can successfully transmit is decreased as the packet size increases. The energy is wasted when the packet collides with PU packets. Secondly, if the packet size is reduced, the ratio of energy consumption in the data transmission slots of Figure 2 to the energy consumption in the access control slot is reduced, which also reduces the energy efficiency. There is a trade-off between the two conditions, and there may be exist an optimal packet size which leads to the best energy efficiency. Sensors could transmit as many packets as possible to improve energy efficiency during their lifetime. In this paper, the metric energy-per-bit(EPB) is used as denoting the ratio of the total energy consumption to the amount of data successfully transmitted. As introduced before, the protocol designed is to minimize the EPB for the network, not only based on an individual condition [45]:

(19) $$\begin{array}{l} P_{j}^{success}=P(idle\;for\;next\;L\;slots|idle\;in\;the\;initial\;slot)\\ \;={\left(1-p_{j}\right)}^{L} \end{array}$$

Every frame needs to consider the adaptation of the packet size because both the PU behavior and sensor activity are time-varying. A CM is awakened and transmits an accessing request message when data needs to be sent. The CH begins to determine the packet size of the data transmission of the current frame according to the sensor activity and the PU behavior when it receives the access request from the CMs. The total energy consumption in the network includes:

◆ The energy consumed by the access control slot, which includes transmitted access request packets and the broadcast of access reply packets received.
◆ The energy consumed of the data transmission slots.

In this work, the star topology is used between each CM and the CH which has an equal distance d. The size of the access request packet is K_1_ bits. The size of the access reply packet is K_2_ bits. In the access control slot, the energy consumption of the whole network is as follows:

(20) $$E^{\mathrm{co}ntrol}=(E_{cir}+\varepsilon d^{2})\times K_{1}+E_{cir}\times K_{2}$$

Data packet transmission refers to the frame structure in Figure 6. A complete data transmission occupies L slots. Eg: the CM i tries to transmit a packet whose length is L slots using channel j. The energy consumption is $E^{tr}(L)$. Let P_j_(l) denote the transmission probability of the collision between the CM and PU. The CM only sends for l<L slots on channel j. P_j_(l) can be expressed as:

(21) $$P_{j}(\text{l})={\left(1-p_{j}\right)}^{l-1}p_{j}\;1\leq l\leq L$$

where p_j_ is the transition probability from idle to busy. The successful transmission probability of a package with the length of L in the slots is $P_{j}^{success}={\left(1-p_{j}\right)}^{L}$. According to the above equations, the anticipated energy consumption of the CM i transmitting data on the channel j is derived as follows:

(22) $${\bar{E}}_{ij}^{data}=\sum_{l=1}^{L}E_{i}^{tr}(l)P_{j}^{l}+E_{i}^{tr}(L)P_{j}^{success}$$

${\bar{E}}_{ij}^{data}$ is not related to i because CM and CH is the same distance, so ${\bar{E}}_{ij}^{data}={\bar{E}}_{j}^{data}$. The probability of successful transmission only depends on the PU behavior, so the expected amount of successful transmissions in channel j is ${\bar{E}}_{ij}^{data}={\bar{E}}_{j}^{data}$.

If the number of available channels is more than the number of active CMs, then less channels are selected to reducs the probability of collisions with other PUs during the data transmission, This is because when p_j_ is less, these channels can easily remain idle if they feel idle in the first slot. N_active_ is on behalf of selected the available channels.

The optimal packet size in terms of number of slots is obtained by, $L_{opt}=\underset{L\in [L_{\min},L_{\max}]}{\arg\min\;}\;EPB$, $L_{\min}$ (the minimum packet size) depends on the MAC frame format of a specific network. $L_{\max}$ (the maximum packet size) is generally selected as the maximum transmission unit (MTU) which is allowed by the network to avoid packet fragmentation. Because an active CM and the available channel change over time, packet size should be adaptive to change, to minimize the EPB used in the current frame of the network. The CH keeps tracking the changes of channel states by interval time between ACK and join by the residual energy balance from CMs to make a decision on the packet size at the beginning of each frame [46,47,48].

### 4.3. Uniform Exploration and Bounded Knapsack-Based Exploitation

Recalling our setting, μi are known *a priori*. In view of this, the agent explores these values by repeatedly drawing a specific arm to get the expected return. However, the agent usually cannot maximize the total expected reward value if it is only focused on exploration, On the contrary, it may be unable to find the optimal arms to draw, if it stops exploring too fast. In view of this, the most important challenge of the bounded MABs is to find an effective balance between exploration and exploitation. We proposed a new algorithm, which can effectively balance exploration with exploitation in this section,. The intuition behind its significant difference is the setting of the ε value to control the degree of exploration. It is very useful for the theoretical analysis. In addition, the method was proved to be effective in many practical applications, relative to other bandits on the basis of methods such as UCB or ε- greedy. In the following, we first introduce the algorithm of the exploring stage, followed by its exploitation phase.

#### 4.3.1. Uniform Exploration

We commit ε part of the budget B to get the expected return value of the arms in the exploration stage. At first, we draw all arms in the first $\left\lfloor \frac{\varepsilon B}{\sum_{i=1}^{N}c_{i}}\right\rfloor$ time steps repeatedly. That is, $S(t)=\{1,...,N\}if1\leq t\leq \left\lfloor \frac{\varepsilon B}{\sum_{i=1}^{N}c_{i}}\right\rfloor$. Then, we can order the arms in ascending cost, and starting with the lowest arm cost, one after another draw the arm, until the next which will be more than the rest of the budget, and then the algorithm repeats the last step, until there is no remaining budget to pull any arms. In view of this, if $x_{i}^{\exp lore}$ is the times of drawing machine arm in the exploration phase, then $\left\lfloor \frac{\varepsilon B}{\sum_{i=1}^{N}c_{i}}\right\rfloor \leq x_{i}^{\exp lore}$. For simplicity, we assume that $L_{i}\geq x_{i}^{\exp lore}$. Otherwise, once L_i_ is reached, we stop drawing arm i. The reason for choosing this method is that we don’t know that which one is the optimal arm in the exploitation stage, so all arms should be drawn equally in the exploration stage.

#### 4.3.2. The Exploitation Stage of the Bounded Knapsack

In the section, we mainly introduce the development phase bounded ε-first algorithm. To do that, we first introduce the bounded knapsack problem that forms the basis of this phase of the method applied. Then, an efficient approximate way to solve the knapsack-based problem that used in the development state is introduced.The bounded knapsack-based problem is modeled as follows. In case of N types of elements, each type i has a length value *v_i_*, and weight *w_i_*. In another way, there is also a knapsack-based with capacity of weight C. A bounded knapsack-based problem selecting the type of integer unit knapsack to maximize the total value of goods, the total weight of such elements is no more than the capacity of the knapsack weight, but each element i can’t be selected not limited to *L_i_* times. Because the goal is to find the non-negative integers $x_{1},x_{2},x_{3},\cdots ,x_{n},\cdots$, that:

(23) $$\max\sum_{i=1}^{N}x_{i}v_{i}\;s.t.\;\sum_{i=1}^{N}x_{i}v_{i}\leq C,\;\forall i：0\leq x_{i}\leq L_{\text{i}}$$

It is important to set each set *L_i_* = 1 then we obtain the standard model based on 0–1 knapsack. Because the limited of the knapsack is a typical NP-hard problem, the optimal solution method does not guarantee a lower cost. However, approximate optimal ways have been used to solve the problem, for example, a bounded greed algorithm. We use a simple effective approximation bounded greedy algorithm, whose computational complexity is O(NlogN) where N is the number of types. This selection reason is that it is used in addition to the efficiency of the theoretical analysis.

The working principle of the bounded greedy algorithm is as follows: Let viwi denote the density. In the early stages, we classify the item types by reducing the density. The computational complexity is O(NlogN). In the first round of the algorithm, we determine the highest density of the item type. In the capacity of no more than a knapsack or item limit L_i_, we select a viable item as more units. After that, we identify with the rest of the feasible elements which are still suitable for the remaining capacity of the knapsack. When it do not exceed the residual capacity of knapsack or the corresponding item, once again we choose as many units as is feasible. We do this step again and again, until no feasible project is left. Obviously, N is the maximum number of rounds. This algorithm is chosen because it can effectively use the theoretical analysis.

Now, we use the bounded knapsack problem to reduce the exploitation stage of the task assignment problem. Let $\overset{\wedge }{\mu }$ represent the estimate value of μi after the exploration stage. The expected value should be calculated by the average samples of the received return of machine arm i. Given this, our goal is to settle the following integer problem:

(24) $$\begin{array}{l} \max\sum_{i=1}^{N}\overset{\wedge }{\mu }x_{i}^{\exp loit}\;s.t.\;\sum_{i=1}^{N}c_{i}x_{i}^{\exp loit}\leq (1-\varepsilon )B,\;\\ \;\forall i：0\leq x_{i}^{\exp loit}\leq L_{\text{i}}-x_{i}^{\exp lore} \end{array}$$

where $x_{i}^{\exp oit}$ is the decision variable. It represents the times arm i is drawn in the exploitation stage. In order to settle the problem, we apply the abovementioned bounded greedy algorithm. With every $x_{i}^{\exp oit}$ value, the following exploitation algorithm is used now: In each time slot, if drawing arm i, and the number of times is no more than $x_{i}^{\exp oit}$, we draw that arm at t time step. The pseudo code of the algorithm is described in Algorithm 1.

**Algorithm 1** Bounded ε-first algorithm1:**Exploration stage:**2:t = 1; $B^{\exp l}=\varepsilon B$3:**while** drawing is feasible **do**4:draw each arm;5:$B_{t+1}^{\exp l}=\varepsilon B_{t}^{\exp l}-\sum_{k-1}^{N}C_{K}$; t =t +1;6:**end while**7:**while** pulling is feasible **do**8:**if** $B_{t}^{\exp l}<\min_{i}c_{i}$ **then**9:STOP! {pulling is not feasible}10:**end if**11:pull arm i(t), where i(t) =t mod N {choose the subsequent arm to pull};12:$B_{t+1}^{\exp l}=B_{t}^{\exp l}-c_{i}(t)$; t =t +1;13:**end while**14:**Exploitation phase:**


## 5. Performance Analysis

At first, the section originates the limit ε-first algorithm with an upper limit, then we demonstrate that by efficiently revising the value of ε for any given ε value. the upper bound to $O(B^{\frac{2}{3}})$ can be extracted.

### 5.1. Regret Bounds of ε-First with Uniform Exploration Algorithm

Recollect that both A_uni_ and A_greedy_ together compose of sequence A_ε-first_, which is the strategy produced by the bounded ε-first algorithm. The sum of the expected performance of A_uni_ and A_greedy_ can represent the expected reward of the strategy. This is:

$$G^{B}(A_{\varepsilon -first})=G^{\varepsilon B}(A_{uni})+G^{(1-\varepsilon )B}(A_{greedy})$$

Generally speaking, it is assumed that the reward probability distribution of each machine arm can meet with [0,1], at the same time drawing cost c_i_ > 1 for each i. Let $i^{\max}=\;\arg\max j\frac{\mu_{j}}{c_{j}}$, Similarly, let $i^{\min}=\;\arg\min j\frac{\mu_{j}}{c_{j}}$. Furthermore, $c^{\max}=\;\arg\max j\frac{\mu_{j}}{c_{j}}$, and $c^{\min}=\;\arg\min j\frac{\mu_{j}}{c_{j}}$. They are described as follows:

**Theorem 1.** Let 0 < ε, β < 1. Assume that $\varepsilon B\geq \sum_{j=1}^{N}c_{j}$, the capability regret of the bounded ε-first method is the best with at least probability β

(25) $$2+\frac{c_{\min}\mu i^{\max}}{c_{i}^{\max}}+\varepsilon Bd_{\max}+2N(\sqrt{\frac{B(-\ln\frac{1-\sqrt[{N}]{\beta }}{2})\sum_{j=1}^{N}c_{j}}{\varepsilon }})$$

where $d_{\max}=\max_{i\neq j}|\frac{\mu_{i}}{c_{i}}-\frac{\mu_{j}}{c_{j}}|$.

### 5.2. Regret Bounds of ε-First with the Successive Rejects Exploration Algorithm

We recall that the capability of the exploitation stage mainly depends on how exactly the accurate ranking of the denseness of the arms can be computed. This stimulates the uniform distribution, which examines the entire same arm, so that the arm can effectively identify the rank of usage, but as a result of the property of the limited greedy algorithm, in fact performance generally depends merely on the exploitation stage at the highest level arm, rather than a complete ordering, because we may run out of pre-budget as the arm reaches a low level. Therefore, it is not clear whether it should concentrate only on advanced machine arms and is not intended to identify the entire sorting. In view of this, the capability of the modified ε-first algorithm is analyzed, wherein the uniform exploration methods are replaced with other exploration methods not designed to estimate correctly the whole sort order. There are many algorithms for solving this problem. We replace the successive rejects for the uniform exploration method, in order to research whether the capability of bounded *ε*-first can be improved. In the following, we first describe how the successive rejects can adjust to our environment, then we provide a regret theory.

## 6. Simulation and Results

The simulation tool of the bandwidth estimation is the Network Simulator version 2 (NS2). Another is Matlab. The simulation scenarios are like a real time wireless sensor network. Firstly, we use an online method based on ACK interval which is used to probe the available bandwidth. Secondly, we utilize the multi-armed bandit algorithm to find the optimal packet size for achieve better energy efficiency and throughput. The algorithms were simulated for capability evaluation in different channel network states.

### 6.1. Bandwidth Estimation Online Learning

The sender monitors ACKs to estimate the bandwidth. More precisely, the sender uses the ACK reception rate and the information an ACK conveys regarding the amount of data. We assume that sender receives the ACK at *t_k_*, *t_k-1_* is the time the previous ACK was received, *d_k_* bytes have been received at the receiver, so the bandwidth such is that:

$$b_{k}=\frac{d_{k}}{t_{k}-t_{k-1}}$$

**Figure 7:**
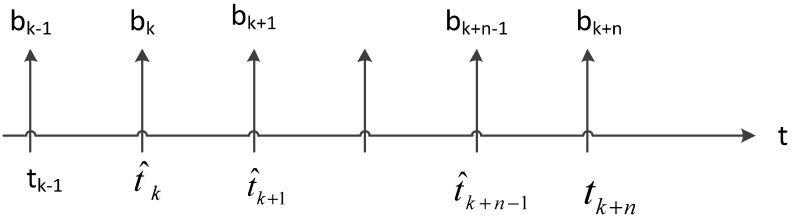
inserting virtual sample.

Due to the effect of the wireless transmission link attenuation or noise, individual ACK time intervals have a greater volatility. Filter logic, which can filter out the noise leading to ACK delay, is very necessary. The choice of filtering logic is very important. According to the experiments, the simple exponential filter cannot effectively filter out the high frequency part. We propose a discrete-time low-pass filter:

(26) $${\hat{b}}_{k}=\frac{\frac{2\tau }{t_{k}-t_{k-1}}-1}{\frac{2\tau }{t_{k}-t_{k-1}}+1}{\hat{b}}_{k-1}+\frac{b_{k}+b_{k-1}}{\frac{2\tau }{t_{k}-t_{k-1}}+1}$$

where, ${\hat{b}}_{k}$ is the effective bandwidth. At *t_k_*, $\frac{1}{\tau }$ is the filtering threshold. This means that all frequency that are higher than $\frac{1}{\tau }$ will be filtered. According to the Nyquist sampling theorem, the sampling period must be less than or equal to $\frac{\tau }{2}$ when the bandwidth sampling is $\frac{1}{\tau }$. As shown in Figure 7, we insert virtual sample values $b_{k+j}=0\;（\text{j}=0,\text{1},\ldots ,\text{n}-\text{1}）$, and when the ACK is not received at *t_k_*, and virtual sampling interval is $\frac{\tau }{m}(m\geq 2)$, namely ${\hat{t}}_{k+j+1}-{\hat{t}}_{k+j}=\frac{\tau }{m}$, ${\hat{t}}_{k+j}$ is the virtual sampling time, when an ACK arrival moment is $t_{k+n}$, $b_{k+n}=\frac{d_{k+n}}{t_{k+n}\;-\;t_{k+n-1}}$ [49,50,51].

**Figure 8:**
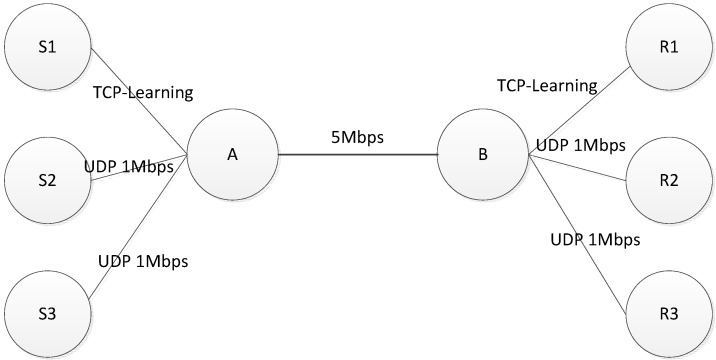
Network topology.

In order to analyze the response time interval and network congestion status, the simulation scenario is shown in Figure 8, where TCP-Learning and the two constant bit rate (CBR) model of UDP share a 5 Mb bottleneck link. Buffer queue is set to 30 packets, and first come first served (FCFS) mode is used.

To analyze the dynamic data model, the TCP–Learning continues to send data over the entire timeline, and the corresponding relation of time and UDP events is shown in Table 2.

**Table 2:** The corresponding relation of time and events.

Time	Events
0 s	TCP–Learning send data
25 s	The first UDP link start sending 1 M constant data
50 s	The second UDP link start sending 1 M constant data
75 s	The second UDP link stop sending
125 s	The second UDP link restart
175 s	The second UDP link stop sending
200 s	The first UDP link stop sending
300 s	TCP-Learning stop sending

The bottleneck bandwidth link is 5 Mb. Link delay is 35 ms. The overall simulation time is 300 s. The parameter settings of the simulation are shown in Table 3.

**Table 3:** Parameter settings.

Type	Value	Type	Value
Bottleneck bandwidth	5 Mb	UDP Transfer rate	1 Mbps
Bottleneck bandwidth delay	35 ms	TCP Packet Size	1500
UDP Packet Size	1500	Total transmission time	300 s
Link error rates	0.0%		

**Figure 9:**
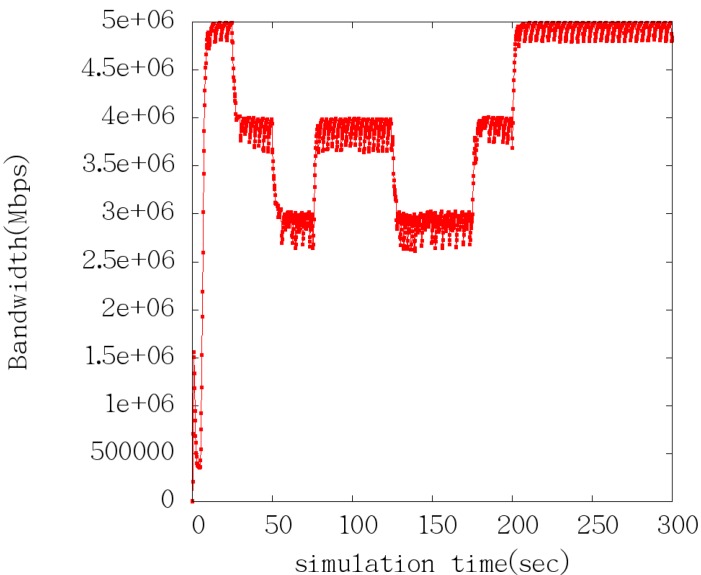
Bandwidth estimation.

**Figure 10:**
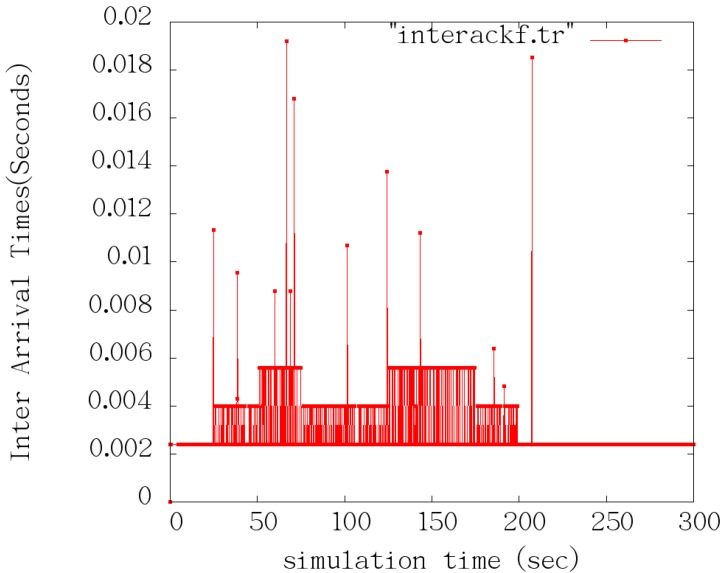
The time interval of ACK.

The simulation results analysis is as follows: according to Table 2, during the 0~25 s, TCP-Learning only sends data in the link. As can be seen from Figure 9 and Figure 10, when the bandwidth is 5 Mb, the time interval of ACK Keep is around 0.0023 s; during the 25~50 s, the first UDP link starts sending 1 Mb constant data, and the bandwidth is 4 Mb, and the time interval of ACK extends about 0.004 s; during the 50~75 s period, the second UDP link starts sending 1 Mb constant data, and the bandwidth becomes 2 Mb, and the time interval of ACK extends about 0.0058 s. As can be seen from the entire simulation process, the ACK interval can better reflect the current situation of the link bandwidth.

### 6.2. The Convergence of MAB

**Figure 11:**
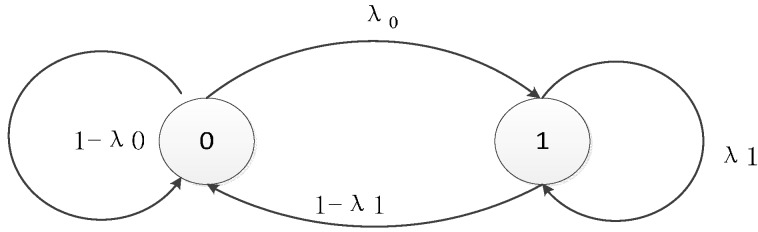
G-E Channel Model.

The simulation is conducted within a cluster of a cognitive radio sensor network. Suppose the authorized users are in the network system, every channel has only two kinds of state S, which are named as two Gilbert-Elliott Markov chain values as shown in Table 4 and Figure 11.

**Table 4:** Parameters of Gilbert-Elliott Markov chains.

Type	Description
S = 1	the current channel free
S = 0	the current state of busy
λ_0_	the channel state transition probability from busy to idle
1 − λ_1_	the channel state transition probability from idle to busy

The whole wireless network simulation parameters are shown in Table 5.

**Table 5:** all the parameters used in the simulation [52,53,54,55].

Parameters	Value
Number of sensors (N)	30
Number of channels (M)	20
Sensor active probability	0.3
Channel transition probability	(0.1)
Slot duration (ts)	4 ms
Data transmission rate (B)	40 kbps
CM control packet size (L)	17 bytes
CH broadcast packet size (L)	20 bytes
CM data packet size (L)	20~128 bytes
Sensor initial energy (*E_in_*)	1 J
RF circuit energy consumption (*E_cir_*)	50 nJ/bit
Amplifier energy required at CH (*ε*)	100 PJ/bit
Distance between sensor and CH (d)	25 m on average

**Figure 12:**
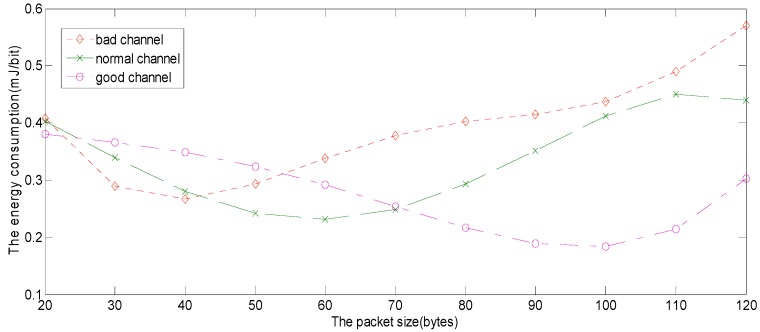
The optimal packet size in the different channel states.

Because the frame error rate is dependent on the packet size, the number of data sent according to the channel quality at any time should be revised. In Figure 12, the channel quality is divided into good channel, normal channel and bad channel. When the wireless network quality is bad, the optimal packet size is 40 bytes. When the channel quality is normal, the optimal packet size is 60 bytes. When the channel quality is good, the optimal packet size is 100 bytes. When the optimal packet size is transmitting and receiving, the energy consumption is the least. It can be concluded that there is always the optimal packet size for any channel state.

**Figure 13:**
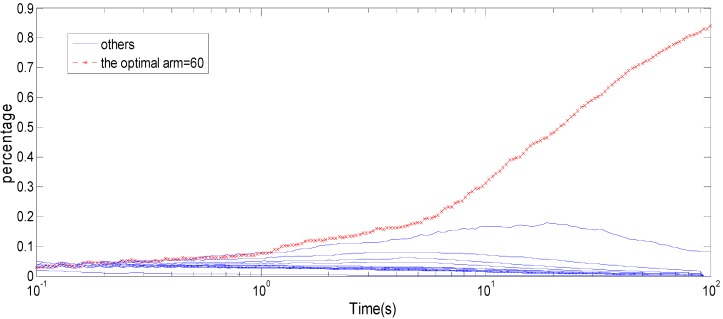
The optimal arm for the same wireless network quality.

The paper maps packet size adaptation to the different arms. When the channel transmission probability is unknown, the challenge of searching the optimal packet size strategy is that the arm is infinite. In order to solve this problem, this paper makes use of sender monitoring ACKs to estimate the bandwidth. When the channel quality is bad, the packet size is between 20 and 50. When the channel quality is normal, the packet size is between 50 and 90. When the channel quality is good, the packet size is between 90 and 120. Figure 13 shows he capability of all arms with the normal wireless network quality for the multi-armed bandits algorithm.

**Figure 14:**
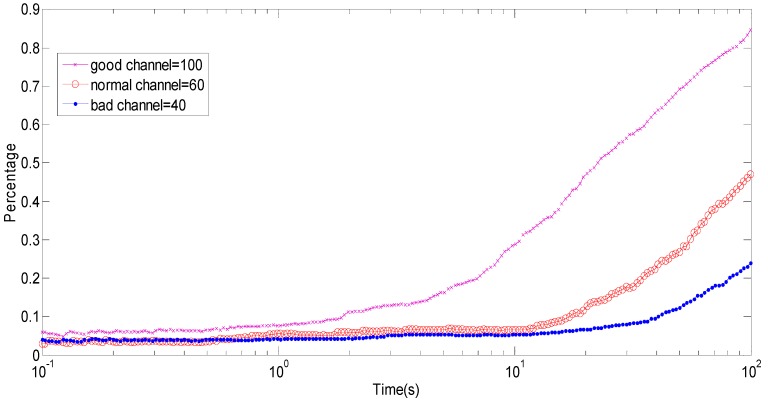
The optimal arm for the different wireless network quality.

The arm 60 is the optimal arm for the wireless network quality, and arm 60 is chosen to reach to 100% with increasing implementation time, otherwise the other arm chosen reaches 0%. The same method can obtain the corresponding optimal arm under the good and bad wireless network quality conditions. As shown in Figure 14, the convergence of the corresponding optimal arm can be obtained with different wireless network quality. As seen in the figure, as the implement time increases, the optimal arm was chosen to run longer then gradually reaches 100%.

### 6.3. Packet Size Adaption Performance

**Figure 15:**
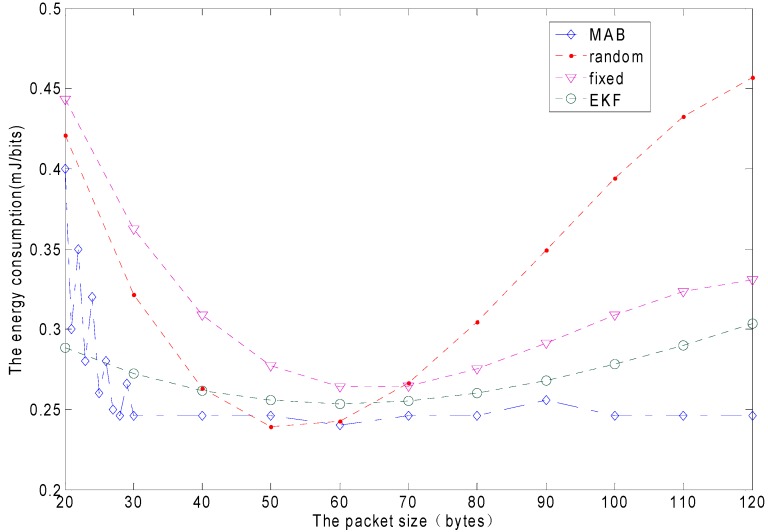
The energy consumption comparison for the different packet size schemes.

The proposed packet size adaptation scheme is examined by comparing it with the random packet size, the fixed packet size and the EKF. Figure 15 shows the comparison results. It is the accumulative network energy efficiency. The network energy efficiency is characterized in terms of energy-per-bit (EPB) which denotes the ratio of the total energy consumption and the amount of data successfully sent. We observe that the MAB packet size adaptation scheme achieves the best energy efficiency across the whole duration of the simulation. The proposed packet size adaptation scheme keeps its energy-per-bit at the lowest level among all packet-sizing schemes. The blue horizontal line represents the MAB adaptive packet size. The EPB is 0.246 mJ/bit. The amount of data successfully sent is 122 Mb. The red line represents the fixed packet sizing scheme, whose energy-per-bit value first decreases, and then it keeps increasing as the packet size increases. The optimal packet size of the fixed scheme is 60 bytes. The energy-per-bit is 0.253 mJ/bit. The amount of data successfully sent is 119 Mb. The purple line represents the random packet sizing scheme, which is still worse than the adaptive packet size performance since the packet size adaptation scheme dynamically tracks the channel behavior . The MAB adaptive packet size is clearly better than the EKF scheme.

**Figure 16:**
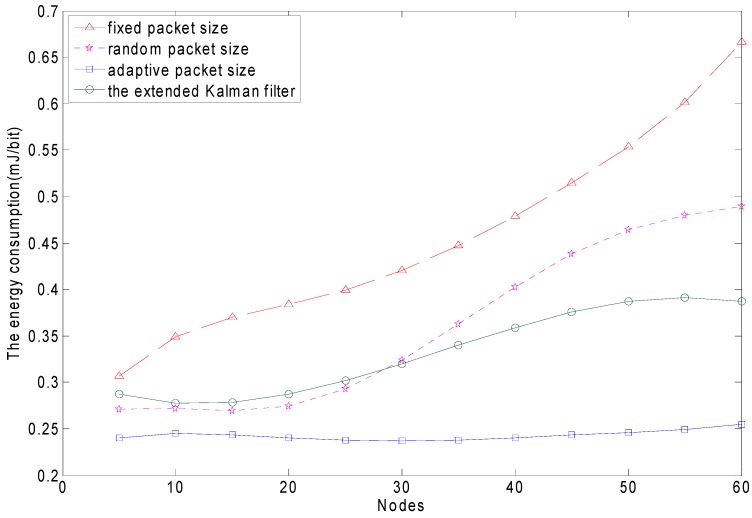
The energy consumption comparison of the different packet size schemes per number of mobile nodes.

The whole network has a cluster head and sensor nodes. The sensor nodes periodically send their sensing data to the cluster head. The sensor nodes are battery operated. The cluster heads usually supply power. As mentioned, the energy can be optimized due to the optimal packet size. Figure 16 shows the comparison of the energy consumption using in the MAB policy, the random packet size, the fixed packet size policy and the EKF policy. The blue horizontal line represents the MAB adaptive packet size. When the mobile node increases, the energy consumption almost does not increase. The red line represents the fixed packet sizing scheme. The energy consumption keeps increasing as the nodes increase. The purple line represents the random packet sizing scheme. The energy consumption is similar to that of the fixed packet sizing scheme. Due to the increasing number of mobile nodes, the probability of conflict increases and the channel quality is poorer. Therefore, as the packet length increases, the energy consumption experiences a sharp rise. The green line represents the EKF scheme. The energy consumption increases slowly, and finally tends to be stable as the packet length increases. The MAB adaptive packet size is clearly better than the other three strategies as the number of mobile nodes increases.

**Figure 17:**
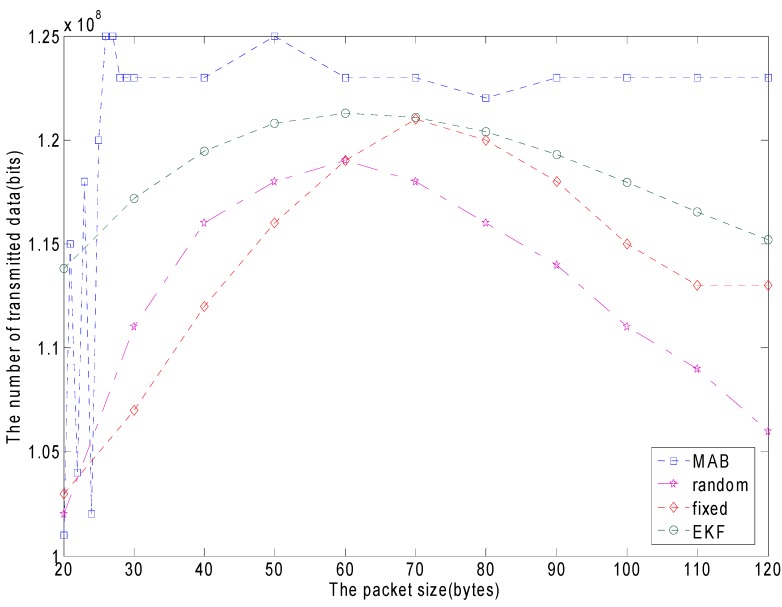
The comparison of the number of successfully transmitted data among different packet-sizing schemes as the packet size changing.

Figure 17 demonstrates the comparison of the amount of transmitted data in the MAB policy, the random packet size and the fixed packet size policy as the packet size is changing. The blue line represents the MAB adaptive packet size. When the channel quality is changed, the packet size is adaptive. In the beginning, the transmitted data is at the jitter stage, since it is searching for the optimal arm according to the MAB strategy at the learning stage, then the amount of transmitted data tends to a constant value once the optimal arm is found. It can be seen that the transmitted data of the MAB policy is significantly better than that of the random, the fixed packet size scheme and the EKF scheme. The reasons for this improvement can be attributed to the adaptive property of the MAC. When the wireless network quality is bad, it reduces the packet size, and reduces the retransmission frequency. In this way errors are reduced and the channel capacity utilization is increased.

**Figure 18:**
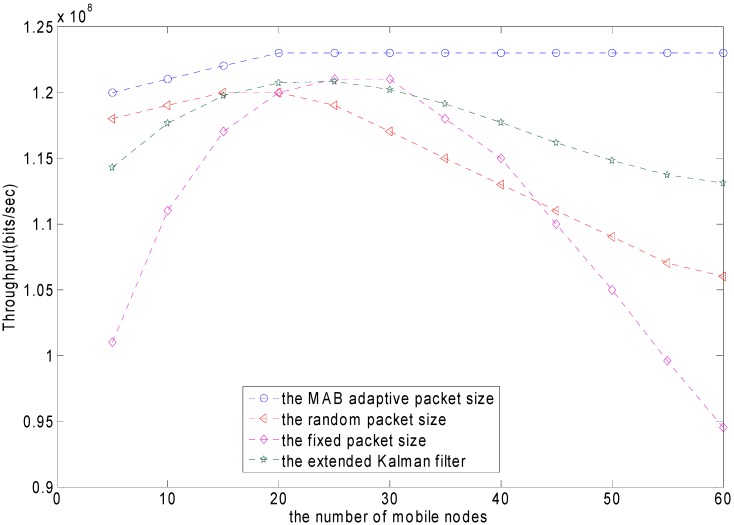
The comparison of the throughput among different packet-sizing schemes as the number of mobile nodes is increasing.

Figure 18 shows the comparison of the throughput with the MAB policy, the random packet size, the fixed packet size policy and the EKF policy as the number of mobile nodes is increasing. The blue line represents the MAB adaptive packet size. When the number of mobile nodes increases, the throughput slowly increases. The red line represents the fixed packet sizing scheme. The purple line represents the random packet sizing scheme. The throughput sharply declines as the nodes increase in number. Due to the increasing number of mobile nodes, the probability of conflicts increases and the channel quality is poorer, therefore, the throughput experiences a sharp drop. The green line represents the EKF. When using the EKF, the throughput slowly decreases as the number of mobile nodes increases.The throughput of the MAB adaptive packet size is clearly better than the other three strategies as the number of mobile nodes is increasing.

### 6.4. Residual Energy Balanceing Channel Assignment

**Figure 19:**
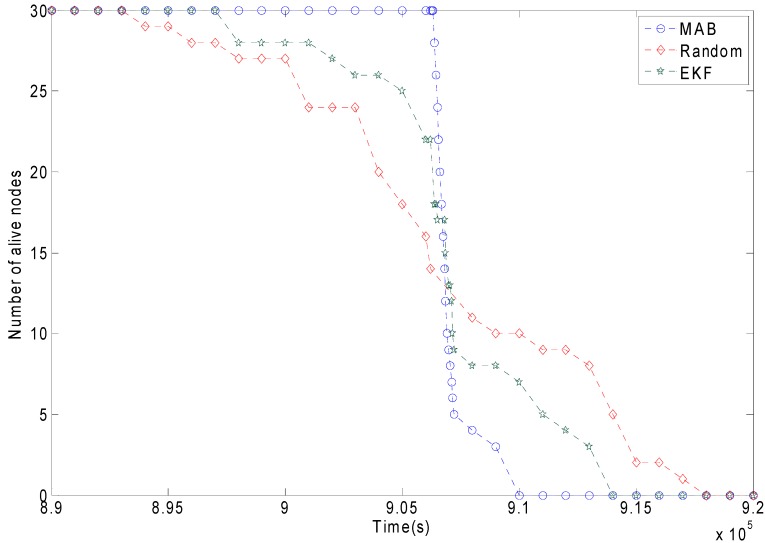
The comparison of the network lifetime between different packet-sizing schemes.

Another type of simulation result is the network residual energy balance. Figure 19 shows the comparison of the network lifetime with the MAB policy, the random scheme and the EKF. Figure 19 verifies the design goal of the residual energy balancing channel assignment is met. The red line represents the random scheme. some nodes run out of battery power after 8.95 × 10^5^ s. A considerable part of the nodes run out of battery after 9.075 × 10^5^ s. Although some nodes still have power after 9.075 × 10^5^ s, since most of the nodes have run out of battery it means the whole network almost cannot run. The blue line represents the MAB adaptive packet size. Before 9.075 × 10^5^ s, almost no nodes fail, and the entire network can run well. The death of sensors is distributed in a narrow time period. This is desirable because the total unused energy is reduced when the network dies. The MAB scheme can maintain the network residual energy balance. The green line represents the EKF. The EKF is slightly better than the random scheme regarding the network residual energy balance. The residual energy balancing effect of the MAB is clearly better than the random scheme and the EKF.

## 7. Conclusions

An adaptive packet size strategy for the energy efficiency of wireless sensor networks is proposed in this paper. The main goal is to reduce the power consumption and extend the whole network life. In order to reach this goal, the paper introduced the bounded MAB to find the optimal packet size to transfer through formulating different packet sizes for different arm under the channel condition. At the same time, in order to achieve fast convergence, we consider the bandwidth evaluation according to ACK. The simulation results demonstrate that the packet size is adaptive when the channel quality changes and the convergence of the corresponding optimal arm can obtain different wireless network quality, as the implementation time increases; the optimal arm chosen to run longer then gradually reaches 100%. We observe that the goodput and energy consumption of the MAB algorithm is much better than the fix frame size and random frame size algorithms under different channel quality scenarios. At the same time it extends the whole network life. In the future, we will consider the cross layer and Partially Observable Markov model to improve the overall performance.

## References

[1] Stankovic JA., Abdelzaher T.F., Lu C., Sha L., Hou J.C. (2003). Real-time communication and coordination in embedded sensor etworks. IEEE Proc..

[2] Chong C., Kumar S. (2003). Sensor networks: Evolution, opportunities,and challenges. IEEE Proc..

[3] Ci S., Sharif H., Nuli K. (2005). Study of an Adaptive Frame Size Predictor to Enhance Energy onservation in Wireless Sensor Networks. IEEE J. Sel. Areas Commun..

[4] Li X., Wang D. (2014). Dynamic spectrum access with packet size adaptation and residual energy balancing for energy-constrained cognitive radio sensor networks. J. Netw. Comput. Appl..

[5] Lucas Martínez N., Martínez J.-F., Hernández Díaz V. (2014). Virtualization of Event Sources in Wireless Sensor Networks for the Internet of Things. Sensors.

[6] Chung E.Y., Benini L., Bogliolo A., Lu Y.H., Micheli G.D. (2002). Dynamic power management for nonstationary service requests. IEEE Trans. Comput..

[7] Gao S, Qian L, Vaman D.R. (2009). Distributed energy efficient spectrum access in cognitive radio wireless ad hoc networks. IEEE Trans. Wirel. Commun..

[8] Chen Y., Zhao Q. (2005). On the lifetime of wireless sensor networks. IEEE Commun. Lett..

[9] Wang Q., Hempstead M., Yang W. A realistic power consumption model for wireless sensor network devices. Proceedings of the Third Annual IEEE Communications Society Conference on Sensor, Mesh and Ad Hoc Communications and Networks.

[10] Yang J., Ulukus S. (2012). Optimal packet scheduling in an energy harvesting communication system. IEEE Trans. Commun..

[11] Akan O.B., Karli O.B., Ergul O. (2009). Cognitive radio sensor networks. IEEE Netw..

[12] Goh H.G., Kwong K.H., Shen C., Michie C., Andonovic I. CogSeNet: A concept of cognitive wireless sensor network. Proceedings of 2010 7th IEEE consumer communications and networking conference (CCNC).

[13] Vijayand G., Bdira E.B.A., Ibnkahla M. (2011). Cognition in wireless sensor networks: A Perspective. IEEE Sens. J..

[14] Zhang H., Zhang Z., Chen X., Yin R. Energy Efficient Joint Source and Channel Sensing in Cognitive Radio Sensor Networks. Proceedings of the IEEE International Conference on Communications (ICC 2011).

[15] Maleki S., Pandharipande A., Leus G. (2011). Energy-efficient distributed spectrum sensing for cognitive sensor networks. IEEE Sens. J..

[16] Liang Z., Feng S., Zhao D., Shen X.S. (2011). Delay performance analysis for supporting realtime traffic in a cognitive radio sensor network. IEEE Trans. Wirel. Commun..

[17] Tian X., Tian Z., Pham K., Blasch E., Chen G. (2013). QoS-aware dynamic spectrum access for cognitive radio networks. Proc. SPIE.

[18] Zhu J., Wang J., Luo T., Li S. (2009). Adaptive transmission scheduling over fading channels for energy-efficient cognitive radio networks by reinforcement learning. Telecommun. Syst..

[19] He H., Wang J., Li S. (2013). Strategic learning of cross-layer design for channel access and transmission rate adaptation in energy-constrained cognitive radio networks. J. Inf. Comput. Sci..

[20] Naqvi H., Berber S., Salcic Z. (2010). Energy efficient collaborative communication with imperfect phase synchronization and rayleigh fading in wireless sensor networks. Phys. Commun..

[21] Sankarasubramaniam Y., Akyildiz I., McLaughlin S. Energy efficiency-based packet size optimization in wireless sensor network. Proceedings of 1st IEEE International Workshop on Sensor Network Protocols and Applications.

[22] Mastronarde N., Schaar M.V.D. (2011). Fast reinforcement learning for energy-efficient wireless communication. IEEE Trans. Signal Process..

[23] Woo A., Culler D. A transmission control scheme for media access in sensor networks. Proceedings of the 7th annual international conference on Mobile computing and networking.

[24] Ho C.J., Vaughan J.W. On line task assignment incrowd sourcing markets. Proceedings of the 26th Conference on Artificial Intelligence.

[25] Bubeck S., Munos R., Stoltz G. Pure exploration for multi-armed bandit problems. Proceedings of the Twentieth international conference on Algorithmic Learning Theory.

[26] Audibert J.-Y., Bubeck S., Munos R. Best arm identification in multi-armed bandits. Proceedings of the Twenty-Third Annual Conference on Learning Theory.

[27] Auer P., Cesa-Bianchi N., Fischer P. (2002). Finite-time analysis of the multiarmed bandit problem. Mach. Learn..

[28] Beygelzimer A., Langford J., Li L., Reyzin L., Schapire R. Contextual bandit algorithms with supervised learning guarantees. Proceedings of the Fourteenth International Conference on Artificial Intelligence and Statistics.

[29] Tran-Thanh L., Stein S., Rogers A., Jennings N.R. (2014). Efficient crowdsourcing of unknown experts using bounded multi-armed bandits. Artif. Intell..

[30] Zhang J., Jiang H., Jiang H.S., Chen C. (2014). Energy-Efficient Policy Based on Cross-Layer Cooperation in Wireless Communication. Int. J. Distrib. Sens. Netw..

[31] Chen F., Kwong S. (2009). Multiuser detection using hidden Markov model. IEEE Trans. Veh. Technol..

[32] Huang S., Liu X., Ding Z. (2011). Decentralized cognitive radio control based on inference from primary link control information. IEEE J. Sel. Areas Commun..

[33] Zhou B., Chen Q., Li T.J., Xiao P. (2014). Online Variational Bayesian Filtering-Based Mobile Target Tracking in Wireless Sensor. Netw. Sens..

[34] Pham D.M., Aziz S.M. (2014). A Real-Time Localization System for an Endoscopic Capsule Using Magnetic Sensors. Sensors.

[35] Robbins H. (1952). Some aspects of the sequential design of experiments. Bull. Am. Meteorol. Soc..

[36] Zhong X., Xu C.Z. (2007). Energy-efficient wireless packet scheduling with quality of service control. IEEE Trans. Mobile Comput..

[37] Li R., Liu X., Xie W., Huang N. (2014). Deployment-Based Lifetime Optimization Model for Homogeneous Wireless Sensor Network under Retransmission. Sensors.

[38] Salodkar N., Bhorkar A., Karandikar A., Borkar V.S. (2008). An online learning algorithm for energy-efficient delay-constrained scheduling over a fading channel. IEEE J. Sel. Areas Commun..

[39] Liu Q., Zhou S., Giannakis G.B. (2005). Queueing with adaptive modulation and coding over wireless links: corss-layer analysis and design. IEEE Trans. Wirel. Commun..

[40] Bandyopadhyay S., Saha S., Maulik U., Deb K. (2008). A simulated annealing-based multiobjective optimization algorithm: AMOSA. IEEE Trans. Evolut. Comput..

[41] Mnih V., Szepesvari C., Audibert J. Empirical Bernstein stopping. Proceedings of the 25th International Conference on Machine Learning.

[42] Gungor O., Tan J., Koksal C.E., Gamal H.E., Shroff N.B. Joint power and secret key queue management for delay limited secure communication. Proceedings of the 2010 IEEE INFOCOM.

[43] Tran-Thanh L., Chapman A., de Cote J.E.M., Rogers A., Jennings N.R. Epsilon-first policies for budget-limited multi-armed bandits. Proceedings of the 24th Conference on Artificial Intelligence.

[44] Tran-Thanh L. (2012). Budget-Limited Multi-Armed Bandits. Ph.D. Thesis.

[45] Tran-Thanh L., Stein S., Rogers A., Jennings N.R. Efficient crowdsourcing of unknown experts using multi-armed bandits. Proceedings of 20th European Conference on Artificial Intelligence, ECAI 2012.

[46] Singh S., Jaakkola T., Littman M., Szepesvári C. (2000). Convergence results for single-step on-policy reinforcement learning algorithms. Mach. Learn..

[47] Jiang H., Liu C., Wu C. (2013). Crosslayer parameter configuration for TCP throughput improvement in cognitive radio networks. Acta Phys. Sin.

[48] Chen Y., Zhang S., Xu S., Li G.Y. (2011). Fundamental trade-offs on green wireless networks. IEEE Commun. Mag..

[49] Del-Valle-Soto C., Mex-Perera C., Orozco-Lugo A., Lara M., Galván-Tejada G.M., Olmedo O. (2014). On the MAC/Network/Energy Performance Evaluation of Wireless Sensor Networks: Contrasting MPH, AODV, DSR and ZTR Routing Protocols. Sensors.

[50] Hussain S.I., Hasnab M.O., Alouini M.S. (2012). Performance analysis of selective cooperation with fixed gain relays in Nakagamim channels. Phys. Commun..

[51] Xu G., Jiang H., Liu C., Wang Y. A heuristic evaluation PDS algorithm for energy-efficient delay constrained scheduling over wireless communication. Proceedings of 2012 IEEE International Conference on Communications (ICC).

[52] Xiong N., Jia X., Yang L.T., Vasilakos A.V., Li Y., Pan Y. (2010). A distributed efficient flow control scheme for multirate multicast networks. IEEE Trans. Parallel Distrib. Syst..

[53] Chen Z., Zou H., Jiang H., Zhu Q., Soh Y.C., Xie L. (2015). Fusion of WiFi, Smartphone Sensors and Landmarks Using the Kalman Filter for Indoor Localization. Sensors.

[54] Vermorel J., Mohri M. Multi-armed bandit algorithms and empirical evaluation. Proceedings of the 16th European Conference on Machine Learning.

[55] Zhang H., Li J. (2015). Modeling and dynamical topology properties of VANET based on complex networks theory. AIP Adv..

